# Prevalence trends in the characteristics of patients with allergic asthma in Beijing, 1994 to 2014

**DOI:** 10.1097/MD.0000000000007077

**Published:** 2017-06-02

**Authors:** Dan Mao, Rui Tang, Rui Wu, Hong Hu, Lu Jin Sun, Hong Zhu, Xue Bai, Jing Guo Han

**Affiliations:** a Respiratory Department, Chinese PLA General Hospital; b Department of Allergy, Peking Union Medical College Hospital, Chinese Academy of Medical Sciences & Peking Union Medical College; c Department of Respiratory Medicine, Peking University Third Hospital, Beijing, China.

**Keywords:** allergic asthma, characteristics, clinical, hospitalization, trend

## Abstract

This study aimed to determine the clinical profiles and prevalence trends during 1994 to 2014 among patients with allergic asthma (AA), which is a clinical phenotype of asthma.

We retrospectively analyzed the characteristics of 319 patients who were diagnosed with AA between March 1, 1994 and February 28, 2014 at 3 Beijing centers.

The patients included 155 males and 164 females, and the mean age was 50.86 ± 15.27 years (range 13–86 years). The proportions of asthma attacks in summer and autumn were 60.7% (1994–1999), 61.8% (1999–2004), 56.4% (2004–2009), and 33.1% (2009–2014). The most frequently used medication at home was theophylline (27.9%), which was followed by inhaled corticosteroids (20.38%), inhaled corticosteroids/long-acting beta-2-agonists (10.66%), and leukotriene receptor antagonists (9.4%). The elderly group had the highest rates of summer and autumn attacks, multiple hospitalizations, reduced pulmonary function, smoking history, and positive allergen tests. The middle-aged group had the lowest rates of summer and autumn attacks, and multiple hospitalizations. The youngest group had the lowest rates of reduced pulmonary function, smoking history, and positive allergen tests. The top 5 allergens were dust (9.1%), mites (8.8%), seafood (8.2%), pollen (6.3%), and animal fur (6%). Women were significantly more likely to have a positive allergen test (93 women vs 68 men).

The present study revealed the characteristics of Chinese patients with AA, and allergen-specific differences in sex and age during 1994 to 2014. The use of therapeutic drugs at home remains insufficient.

## 1 Introduction

### 1.1 Background

Asthma is characterized by chronic airway inflammation and diagnosed based on various respiratory symptoms, such as wheezing, shortness of breath, chest tightness, cough, and expiratory airflow limitation.^[1]^ Asthma is the most common chronic pulmonary disease worldwide and has many phenotypes, such as allergic asthma (AA), nonallergic asthma, late-onset asthma, and asthma with fixed airflow limitations. AA is the most common phenotype of asthma, and is associated with allergic disease or a family history of allergies.^[1]^

China is the world's largest developing country and has recently undergone many obvious changes. In addition, the prevalence of AA has been affected by these changes, which involve the traditional lifestyle and living environment. The prevalence of AA increased from 7.3% to 8.4% during 2001 to 2010 in the United States,^[2]^ and from 1.54% to 2.32% during 2000 to 2010 in China.^[3,4]^ Furthermore, acute asthma exacerbation is associated with a substantial health burden, with approximately 2 million emergency visits and 373,000 hospitalizations in 2012.^[5]^ These conditions create significant public health concerns and a heavy economic burden. Thus, a better understanding of the features of AA could help clinicians provide more effective care and reduce the socioeconomic burden.

### 1.2 Objective

To the best of our knowledge, few studies have focused on the characteristics of patients with AA. Therefore, the present study aimed to evaluate the clinical profiles, allergens, and sex and age-specific differences in the characteristics of 319 patients who were diagnosed with AA at 3 Beijing centers between 1994 and 2014.

## 2 Methods

### 2.1 Study design

This multicenter retrospective study evaluated patients from 3 tertiary hospitals in Beijing who were hospitalized between March 1, 1994 and February 28, 2014. The hospitals were the Peking Union Medical College Hospital, the Peking University Third Hospital, and the Chinese PLA General Hospital. We collected data regarding the patients’ general characteristics (age, sex, smoking, and family history), clinical features, allergens, hospitalization history, and medications used at home. One doctor performed the data collection at each hospital. This study's retrospective design was approved by the Ethics Committee of Chinese PLA General Hospital.

### 2.2 Patients

All patients fulfilled the following criteria: age of ≥14 years; a history of at least 1 hospitalization at a participating center during the study period; and had a definitive diagnosis of asthma (history of variable respiratory symptoms and airflow variability), based on the official global guidelines.^[1]^ Airflow variability was defined as having a >12% and 200-mL increase in forced expiratory volume during 1 second (FEV1) after the inhalation of salbutamol (400 μg) or a 20% reduction in FEV1 (<16 mg/mL) after the inhalation of methacholine. Patients were diagnosed with AA based on fulfilling at least 1 of the following conditions: a personal and/or family history of allergic disease (eg, allergic rhinitis, eczema, or food or drug allergy), at least 1 positive result from allergen testing, increased serum levels of immunoglobulin E (IgE), and pretreatment-induced sputum that revealed eosinophilic airway inflammation.^[1]^ Cases with incomplete data were excluded from the study.

### 2.3 Statistical analysis

Continuous variables were reported as mean ± standard deviation, and categorical/binary variables were reported as number and percentage, frequency distribution, or constituent ratio. Differences between the groups were assessed using 1-way analysis of variance (ANOVA) or chi-square tests, as appropriate. Differences were considered statistically significant at a *P* value <.05. Statistical analyses were performed by using IBM SPSS software (version 20.0; IBM Corp., Armonk, NY).

## 3 Results

### 3.1 General information

The 319 eligible patients included 22 cases from the Peking Union Medical College Hospital, 164 cases from the Peking University Third Hospital, and 133 cases from the Chinese PLA General Hospital. The patients had a mean age of 50.86 ± 15.27 years (range 13–86 years) and included 155 males.

### 3.2 Clinical characteristics

The patients were divided according to their hospitalization period: group A (1994.3.1–1999.2.28), group B (1999.3.1–2004.2.29), group C (2004.3.1–2009.2.28), and group D (2009.3.1–2014.2.28). All 4 groups had similar basic characteristics (age, sex, smoking, and family history), and no significant differences were observed (Table 1).

**Table 1:**
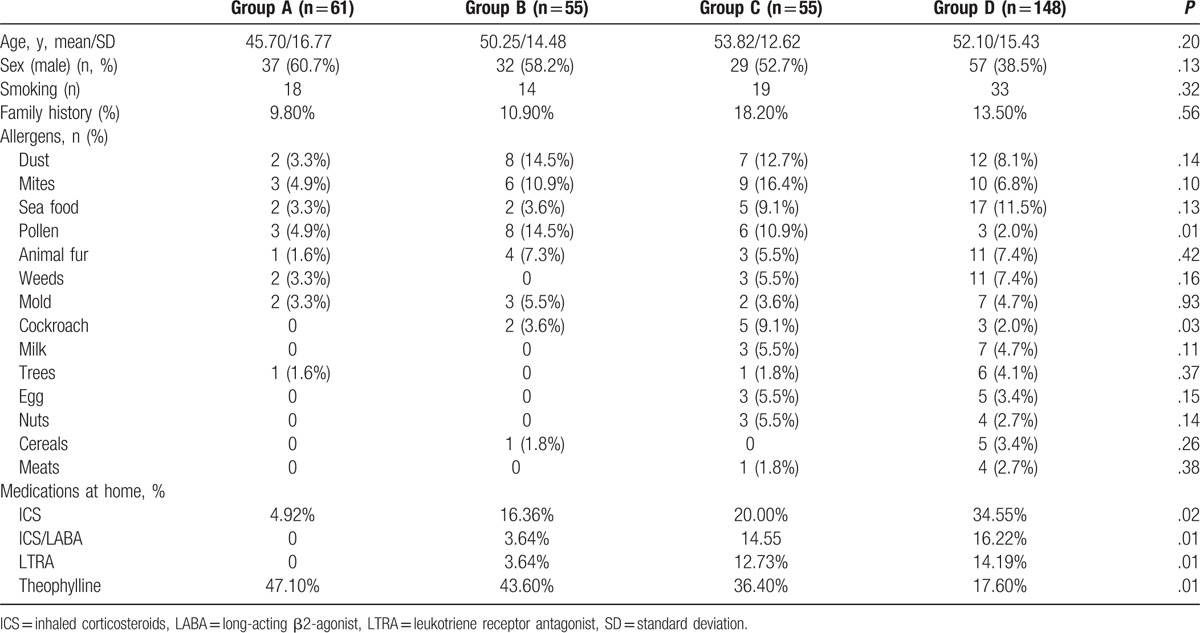
Patient demographics in the 4 groups.

**Figure 1:**
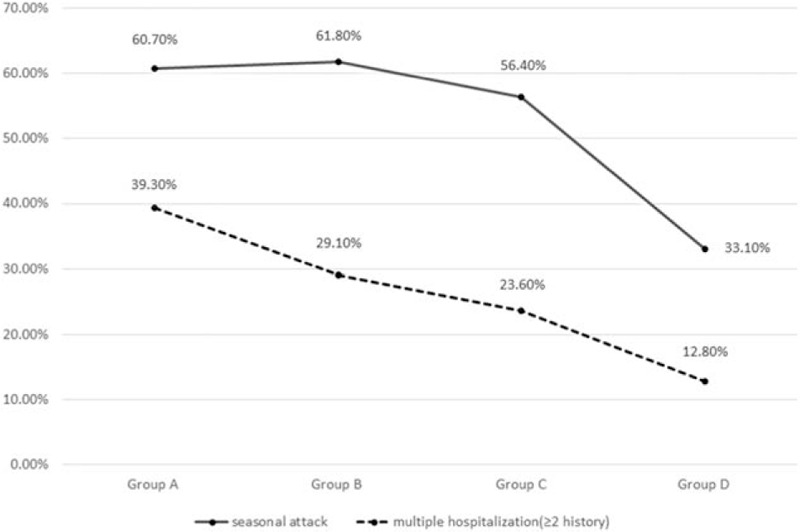
Trends in summer and autumn attacks and multiple hospitalizations.

**Figure 2:**
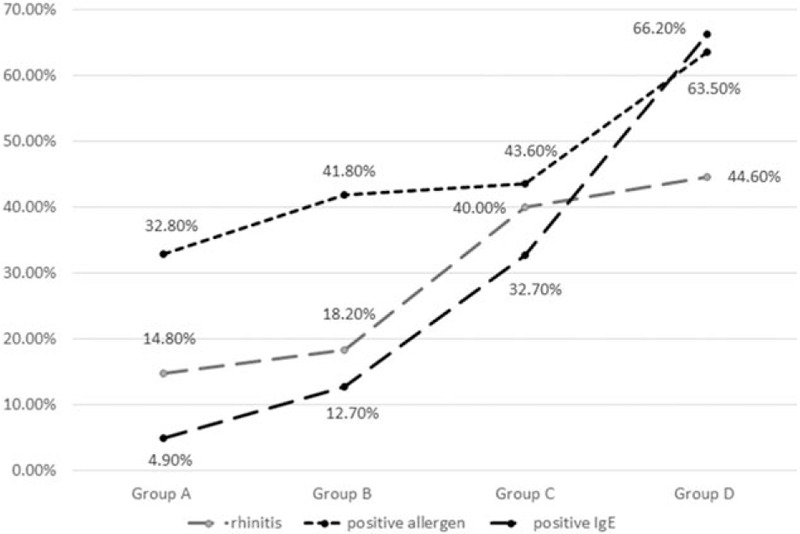
Trends in rhinitis, and positivity to allergens, and immunoglobulin E (IgE).

All 4 groups exhibited general downward trends in the frequencies of summer and autumn attacks (*P* = .02) and multiple hospitalizations (≥2 hospitalizations, *P* = .01) (Fig. 1). All 4 groups also exhibited significant general upwards trends in the frequencies of allergic rhinitis (*P* = .02), positive allergen tests (*P* = .01), and positive IgE tests (*P* = .01) (Fig. 2).

### 3.3 Medications used at home for AA

Four kinds of drugs that were extensively used at home were analyzed and compared (Table 1). All 4 groups exhibited gradually increasing usage proportions for inhaled corticosteroids (ICS) (*P* = .02), ICS/long-acting beta-2-agonist (LABA) (*P* = .01), and leukotriene receptor antagonists (LTRAs) (*P* = .01). A decreasing trend was observed for theophylline (*P* = .01) (Table 1). Group D exhibited the highest percentages for ICS (34.55%), ICS/LABA (16.22%), and LTRA (14.19%), and the lowest percentage for theophylline (17.6%). Among the various drugs, the most frequently used medication at home was theophylline (27.9%), which was followed by ICS (20.38%), ICS/LABA (10.66%), and LTRA (9.4%).

### 3.4 Influence of allergic rhinitis on pulmonary function

Abnormal lung function was defined as <80% for the percentage of the predicted FEV1 value (FEV1%PRED) or a peak expiratory flow (PEF) value of <80%. Patients with AA and with or without allergic rhinitis did not exhibit any significant differences in FEV1%PRED or PEF (Table 2).

**Table 2:**
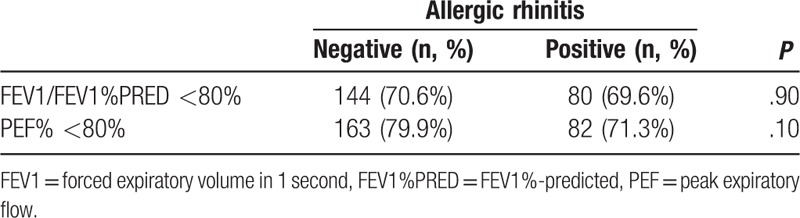
The relationship between allergic rhinitis and pulmonary function.

### 3.5 Allergens

Among the various allergens, the most frequently positive allergen was dust (9.1%), which was followed by mites (8.8%), seafood (8.2%), pollen (6.3%), and animal fur (6%) (Table 3). Significantly differences between groups A, B, C, and D were observed for pollen (*P* = .01) and cockroach (*P* = .03) allergens. No other significant differences were observed (Table 1).

**Table 3:**
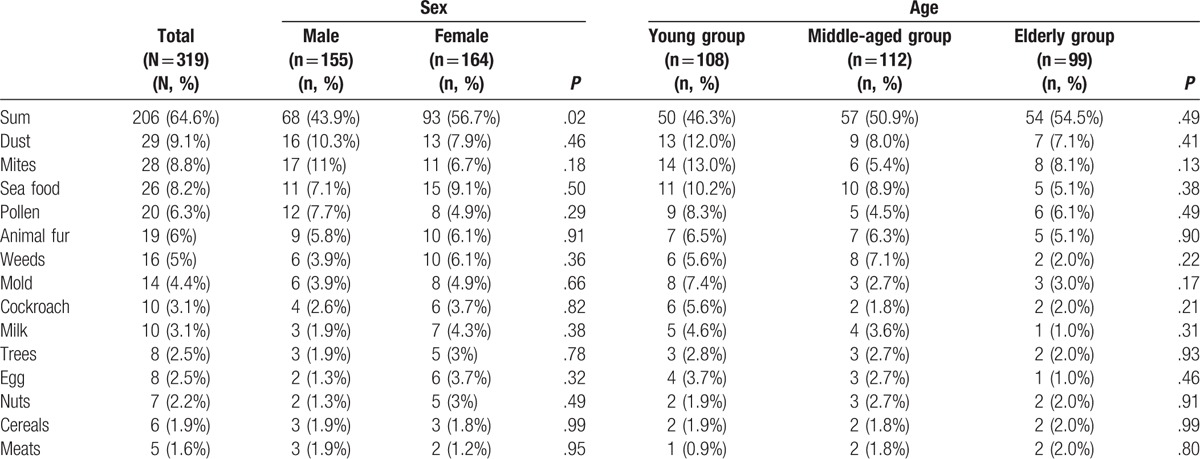
Analysis of allergic asthma allergens.

### 3.6 Sex-specific differences in allergens

Women had significantly more positive allergen tests, compared with men (93 women vs 68 men; *P* < .05). However, the sex-specific proportions of the 14 most common allergens were not significantly different.

### 3.7 Age-specific differences in allergens

The patients were divided into a young group (<45 years old), a middle-aged group (45–60 years old), and an elderly group (>60 years old). The elderly group had the highest proportions for meats and cereals, and the lowest proportions for dust, animal fur, trees, milk, egg, and seafood. The middle-aged group had the highest proportions for weeds and nuts, and the lowest proportions for mold, mites, cockroach, and pollen. The young group had the highest proportions for dust, animal fur, trees, milk, egg, seafood, mold, mites, cockroach, and pollen, but the lowest proportions for nuts and meats (Table 3).

### 3.8 Age-specific differences in clinical characteristics

The elderly group had the highest proportions of summer and autumn attacks (61.6%; *P* = .02), multiple hospitalizations (37.4%; *P* = .01), reduced pulmonary function (FEV1%PRED of <80% = 79.8%; *P* = .04; PEF of <80% = 89.9%; *P* = .01), smoking history (35.4%; *P* = .02), and positive allergen tests (54.5%; *P* = .49). The middle-aged group had the lowest proportions of summer and autumn attacks (39.3%) and multiple hospitalizations (17.1%). The young group had the lowest proportions of reduced pulmonary function (FEV1%PRED of <80% = 65.7%; PEF of <80% = 69.4%), smoking history (18.5%), and positive allergen tests (46.3%) (Tables 3 and 4).

**Table 4:**
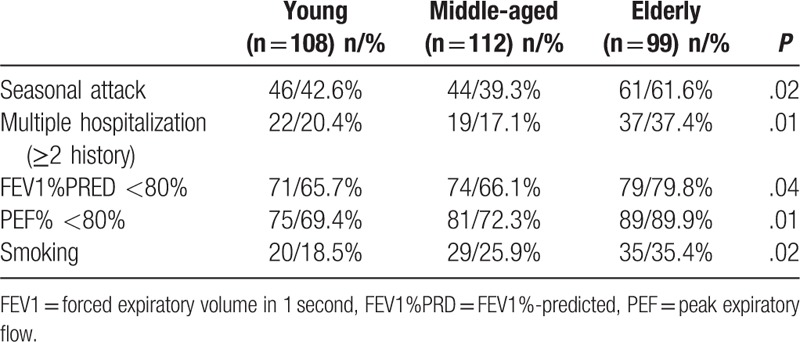
Comparison of the different age groups.

## 4 Discussion

The present study assessed the specific features and prevalence trends of AA in Beijing, and the findings may be useful for developing interventions to prevent and manage AA. Our results indicate that the proportions of summer and autumn AA attacks, reduced pulmonary function, multiple hospitalizations, and rhinitis complications increased during the most recent 10 years (2004–2014), compared with the previous 10 years (1994–2004).

Allergic asthma is usually induced by specific environmental factors or foods during summer or autumn. With the rapid urbanization and industrialization of China, many serious air pollution problems have emerged,^[6]^ and air pollutants have become new AA allergens, which has induced changes in the clinical features of AA. For example, seasonal differences in pollen production are linked to seasonal trends in asthma attacks, and all 4 study groups had significantly positive allergen tests. However, the present study also revealed a gradual decrease in the proportion of seasonal asthma attacks, which may be related to the increased suspension of airborne particles containing allergens, a prolonged pollen season as a consequence of global warming,^[6]^ and modification of the allergen by air pollutants.^[7]^ Moreover, we observed an increasing prevalence of allergic rhinitis, which agrees with the increasing prevalence of AA in a previous report.^[8]^ Previous epidemiological studies of allergic rhinitis have confirmed that approximately 80% of asthma cases involve allergic rhinitis, and 10% to 40% of allergic rhinitis cases also involve asthma.^[9–13]^ These studies concluded that allergic rhinitis may be a risk factor for the development of asthma,^[9]^ and that uncontrolled allergic rhinitis may worsen asthma symptoms, increase bronchial hyperactivity, and reduce lung function.^[13]^ Inverse relationships have also been observed for asthma affecting allergic rhinitis.^[14]^ However, the present study did not detect a clear relationship between uncontrolled allergic rhinitis and reduced lung function, and we hope to perform further studies to examine this issue.

Specific allergens are vital triggers for AA exacerbation, and the most common positive allergens in the present study were dust, mites, seafood, pollen, and animal fur. However, in the United States, the 5 most common allergens are *Alternaria*, *Aspergillus*, *Dermatophagoides pteronyssinus*, *Dermatophagoides farinae*, and cat dander.^[15]^ Among men in the present study, the 5 most common allergens were mites, dust, pollen, seafood, and animal fur, whereas the most common allergens among women were seafood, dust, mites, animal fur, and weeds. These differences suggest that patients with AA exhibit differences according to their race, sex, and age. Thus, Chinese AA allergen data should likely be used to develop local interventions to prevent asthma exacerbation.

In addition to climate change, smoking is also a critical risk factor for the exacerbation and poor control of asthma, and also an impaired corticosteroid response.^[16–18]^ In the present study, we found that elderly patients had the highest proportion of smoking history (35.4%; *P* = .02) and the poorest lung function (FEV1%PRED of <80% = 79.8%; *P* = .04; PEF of <80% = 89.9%; *P* = .01). In this context, impaired lung function may be related to smoking interfering with airway cleaning functions, increasing airway epithelial cell damage, accelerating airway inflammation, increasing the risk of asthma attacks, and reducing sensitivity to glucocorticoid therapy.^[19–23]^ Thus, patients with asthma should stop smoking to improve their quality of life.

Interestingly, we observed that female patients were more likely to have positive allergen test results, compared with male patients. Thus, it appears that women may be more vulnerable to allergens and more susceptible to allergic diseases. Asthma-related morbidities also exhibit age and sex-specific differences. For example, a higher prevalence of AA is observed among boys before puberty (vs girls), although women exhibit a higher prevalence, compared with men.^[24]^ In addition, women are more likely to be hospitalized, compared with men.^[25–27]^ Previous studies have revealed a decreasing trend when middle-aged patients were compared with young patients, although a subsequent increase was observed among elderly patients.^[28]^ In the present study, the elderly group had the highest proportions of multiple hospitalizations and summer and autumn attacks, but the poorest pulmonary function. However, we also observed a gradually increasing trend among middle-aged patients, which indicates that this condition should be monitored in middle-aged patients to reduce any later increase in severity among elderly patients. Furthermore, the sex and age-specific differences that we observed may be useful for developing both preventative and therapeutic interventions, which could be adjusted for the patient's age and sex.

Appropriate medication and education are 2 important factors that influence the successful prevention of asthma exacerbation.^[29]^ ICS reduces asthma symptoms, increases lung function, improves quality of life, and reduces the risks of exacerbation, asthma-related hospitalizations, and death.^[30–33]^ The present study revealed gradually increasing rates of using medications at home (eg, ICS, ICS/LABA, and LTRA), as recommended by the Global Initiative for Asthma guidelines^[1]^ and other relevant bronchial asthma guidelines. However, these increases remain insufficient (ICS: 20.38%, ICS/LABA: 10.66%, and LTRA: 9.4%), and the usage rates are much lower than those in developed countries.^[34]^ These changes are likely influenced by improved treatment adherence among patients with asthma, which is related to reformed education approaches, good doctor/patient partnerships, and an increased level of medical service in the Chinese population.^[35,36]^ However, the medical insurance system may not evenly cover all patients, and socioeconomic status varies greatly between different Chinese cities.^[29]^ Moreover, many patients express worry regarding adverse drug reactions, and ineffective treatment remains a vital cause of asthma exacerbation. Thus, the prevention of asthma attacks may be improved through more appropriate asthma therapy, further improving patient compliance, and providing more culturally and linguistically appropriate asthma education.

### 4.1 Limitations

The present study has several limitations that warrant consideration. First, we only evaluated records from inpatients, as the records for outpatients are often simpler and briefer. Second, we could not analyze the effects of lifestyle and diet on the patients’ changing features, as these details are not included in their inpatient clinical records. Third, the limited sample size and retrospective design are associated with known risks of bias. Thus, large high-quality prospective studies with long follow-ups are needed to confirm and explain the relationship between air pollution and the prevalence of AA.

## 5 Conclusions

In conclusion, the present study revealed several changes in the characteristics of patients with AA during 1994 to 2014. We observed age and sex-specific differences, and also differences between smokers and nonsmokers in the different age groups. Furthermore, the use of therapeutic drugs at home remains insufficient.

## References

[1] 2016 GINA Report, Global Strategy for Asthma Management and Prevention. Available at: http://ginasthma.org/wp-content/uploads/2016/04/wms-GINA-2016-main-report-final.pdf.

[2] Akinbami LJ Moorman JE Bailey C Trends in asthma prevalence, health care use, and mortality in the United States, 2001–2010. NCHS Data Brief 2012:1–8. 22617340

[3] Chen YZ [A nationwide survey in China on prevalence of asthma in urban children]. Chin J Pediatr 2003;41:123–7. 14759318

[4] Asthma. NCGoC. [Third nationwide survey of childhood asthma in urban areas of China]. Chin J Pediatr 2013;51:729–35. 24406223

[5] Hasegawa K Brenner BE Nowak RM Association of guideline-concordant acute asthma care in the emergency department with shorter hospital length of stay: a multicenter observational study. Acad Emerg Med 2016;23:616–22. 26833429 10.1111/acem.12920

[6] D’Amato G Vitale C De Martino A Effects on asthma and respiratory allergy of climate change and air pollution. Multidisciplinary Respir Med 2015;10:39. 10.1186/s40248-015-0036-x PMC4687168 26697186

[7] Ouyang Y Xu Z Fan E Effect of nitrogen dioxide and sulfur dioxide on viability and morphology of oak pollen. Int Forum Allergy Rhinol 2016;6:95–100. 26332319 10.1002/alr.21632

[8] Bousquet J Van Cauwenberge P Khaltaev N Allergic rhinitis and its impact on asthma. J Allergy Clin Immunol 2001;108 (5 suppl):S147–334. 11707753 10.1067/mai.2001.118891

[9] Dogru M Investigation of asthma comorbidity in children with different severities of allergic rhinitis. Am J Rhinol Allergy 2016;30:186–9. 27216348 10.2500/ajra.2016.30.4315

[10] Leynaert B Neukirch F Demoly P Epidemiologic evidence for asthma and rhinitis comorbidity. J Allergy Clin Immunol 2000;106 (5 suppl):S201–5. 11080732 10.1067/mai.2000.110151

[11] Guerra S Sherrill DL Martinez FD Rhinitis as an independent risk factor for adult-onset asthma. J Allergy Clin Immunol 2002;109:419–25. 11897985 10.1067/mai.2002.121701

[12] Higuchi O Adachi Y Itazawa T Rhinitis has an association with asthma in school children. Am J Rhinol Allergy 2013;27:e22–5. 23406593 10.2500/ajra.2013.27.3846

[13] Mener DJ Lin SY Improvement and prevention of asthma with concomitant treatment of allergic rhinitis and allergen-specific therapy. Int Forum Allergy Rhinol 2015;5 (suppl 1):S45–50. 26072703 10.1002/alr.21569

[14] Camargos P Ibiapina C Lasmar L Allergic rhinitis and asthma require an integrated management. Thorax 2012;67:1014 [author reply 1014]. 10.1136/thoraxjnl-2012-201666 22354013

[15] Beck AF Huang B Kercsmar CM Allergen sensitization profiles in a population-based cohort of children hospitalized for asthma. Ann Am Thorac Soc 2015;12:376–84. 25594255 10.1513/AnnalsATS.201408-376OC PMC4418318

[16] Allegra L Cremonesi G Girbino G Real-life prospective study on asthma control in Italy: cross-sectional phase results. Respir Med 2012;106:205–14. 22035853 10.1016/j.rmed.2011.10.001

[17] McLeish AC Zvolensky MJ Asthma and cigarette smoking: a review of the empirical literature. J Asthma 2010;47:345–61. 20528586 10.3109/02770900903556413

[18] Tomlinson JE McMahon AD Chaudhuri R Efficacy of low and high dose inhaled corticosteroid in smokers versus non-smokers with mild asthma. Thorax 2005;60:282–7. 15790982 10.1136/thx.2004.033688 PMC1747368

[19] Jarvis D Chinn S Luczynska C The association of smoking with sensitization to common environmental allergens: results from the European Community Respiratory Health Survey. J Allergy Clin Immunol 1999;104:934–40. 10550735 10.1016/s0091-6749(99)70071-0

[20] Polosa R Thomson NC Smoking and asthma: dangerous liaisons. Eur Respir J 2013;41:716–26. 22903959 10.1183/09031936.00073312

[21] Siroux V Pin I Oryszczyn MP Relationships of active smoking to asthma and asthma severity in the EGEA study. epidemiological study on the Genetics and Environment of Asthma. Eur Respir J 2000;15:470–7. 10759439 10.1034/j.1399-3003.2000.15.08.x

[22] Stapleton M Howard-Thompson A George C Smoking and asthma. J Am Board Fam Med 2011;24:313–22. 21551404 10.3122/jabfm.2011.03.100180

[23] Nagasaki T Matsumoto H Influences of smoking and aging on allergic airway inflammation in asthma. Allergol Int 2013;62:171–9. 23612496 10.2332/allergolint.12-RA-0523

[24] Postma DS Gender differences in asthma development and progression. Gender Med 2007;4 (suppl B):S133–46. 10.1016/s1550-8579(07)80054-4 18156099

[25] Woods SE Brown K Engel A The influence of gender on adults admitted for asthma. Gender Med 2010;7:109–14. 10.1016/j.genm.2010.03.005 20435273

[26] Prescott E Lange P Vestbo J Effect of gender on hospital admissions for asthma and prevalence of self-reported asthma: a prospective study based on a sample of the general population. Copenhagen City Heart Study Group. Thorax 1997;52:287–9. 9093349 10.1136/thx.52.3.287 PMC1758523

[27] Trawick DR Holm C Wirth J Influence of gender on rates of hospitalization, hospital course, and hypercapnea in high-risk patients admitted for asthma: a 10-year retrospective study at Yale-New Haven Hospital. Chest 2001;119:115–9. 11157592 10.1378/chest.119.1.115

[28] Zein JG Udeh BL Teague WG Impact of age and sex on outcomes and hospital cost of acute asthma in the United States, 2011–2012. PloS One 2016;11:e0157301. 27294365 10.1371/journal.pone.0157301 PMC4905648

[29] Su N Lin J Chen P Evaluation of asthma control and patient's perception of asthma: findings and analysis of a nationwide questionnaire-based survey in China. J Asthma 2013;50:861–70. 23713625 10.3109/02770903.2013.808346

[30] O’Byrne PM Barnes PJ Rodriguez-Roisin R Low dose inhaled budesonide and formoterol in mild persistent asthma: the OPTIMA randomized trial. Am J Respir Crit Care Med 2001;164 (8 Pt 1):1392–7. 11704584 10.1164/ajrccm.164.8.2104102

[31] Pauwels RA Pedersen S Busse WW Early intervention with budesonide in mild persistent asthma: a randomised, double-blind trial. Lancet (London, England) 2003;361:1071–6. 10.1016/S0140-6736(03)12891-7 12672309

[32] Adams NP Bestall JB Malouf R Inhaled beclomethasone versus placebo for chronic asthma. Cochrane Database Syst Rev 2005;CD002738. 15674896 10.1002/14651858.CD002738.pub2 PMC8447862

[33] Suissa S Ernst P Benayoun S Low-dose inhaled corticosteroids and the prevention of death from asthma. N Engl J Med 2000;343:332–6. 10922423 10.1056/NEJM200008033430504

[34] Haahtela T Tuomisto LE Pietinalho A A 10 year asthma programme in Finland: major change for the better. Thorax 2006;61:663–70. 16877690 10.1136/thx.2005.055699 PMC2104683

[35] Poureslami I Nimmon L Doyle-Waters M Effectiveness of educational interventions on asthma self-management in Punjabi and Chinese asthma patients: a randomized controlled trial. J Asthma 2012;49:542–51. 22715910 10.3109/02770903.2012.682125

[36] Hilvering B Vijverberg SJ Jansen J Diagnosing eosinophilic asthma using a multivariate prediction model based on blood granulocyte responsiveness. Allergy 2016;[Epub 2016/12/29]. doi: 10.1111/all.13117. PubMed PMID: 28029172. 10.1111/all.13117 28029172

